# Bioavailability of Cd, Zn and Hg in Soil to Nine Recombinant Luminescent Metal Sensor Bacteria

**DOI:** 10.3390/s8116899

**Published:** 2008-11-04

**Authors:** Olesja Bondarenko, Taisia Rõlova, Anne Kahru, Angela Ivask

**Affiliations:** Laboratory of Molecular Genetics, National Institute of Chemical Physics and Biophysics, Akadeemia tee 23, 12618, Tallinn, Estonia; E-mails: olesja.bondarenko@kbfi.ee; taisia.rolova@uku.fi; anne.kahru@kbfi.ee

**Keywords:** biosensor, hazard assessment, Gram-positive, Gram-negative, *Escherichia*, *Pseudomonas*, *Staphylococcus*, *Bacillus*

## Abstract

A set of nine recombinant heavy metal-specific luminescent bacterial sensors belonging to Gram-negative (*Escherichia* and *Pseudomonas*) and Gram-positive (*Staphylococcus and Bacillus*) genera and containing various types of recombinant metal-response genetic elements was characterized for heavy metal bioavailability studies. All nine strains were induced by Hg and Cd and five strains also by Zn. As a lowest limit, the sensors were detecting 0.03 μg·L^-1^ of Hg, 2 μg·L^-1^ of Cd and 400 μg·L^-1^ of Zn. Limit of determination of the sensors depended mostly on metal-response element, whereas the toxicity of those metals towards the sensor bacteria was mostly dependent on the type of the host bacterium, with Gram-positive strains being more sensitive than Gram-negative ones. The set of sensors was used to evaluate bioavailability of Hg, Cd and Zn in spiked soils. The bioavailable fraction of Cd and Zn in soil suspension assay (2.6 – 5.1% and 0.32 – 0.61%, of the total Cd and Zn, respectively) was almost comparable for all the sensors, whereas the bioavailability of Hg was about 10-fold higher for Gram-negative sensor cells (30.5% of total Hg), compared to Gram-positive ones (3.2% of the total Hg). For Zn, the bioavailable fraction in soil-water suspensions and respective extracts was comparable (0.37 *versus* 0.33% of the total Zn). However, in the case of Cd, for all the sensors used and for Hg concerning only Gram-negative sensor strains, the bioavailable fraction in soil-water suspensions exceeded the water-extracted fraction about 14-fold, indicating that upon direct contact, an additional fraction of Cd and Hg was mobilized by those sensor bacteria. Thus, for robust bioavailability studies of heavy metals in soils any type of genetic metal-response elements could be used for the construction of the sensor strains. However, Gram-positive and Gram-negative senor strains should be used in parallel as the bioavailability of heavy metals to those bacterial groups may be different.

## 1. Introduction

Heavy metals released into the environment both from natural and anthropogenic sources accumulate in soils and sediments, thus creating polluted environments and posing potential risk to soil organisms. Most of the soil or sediment-accumulated heavy metals tend to become tightly sorbed to soil/sediment solid matrix [1]. Indeed, it has been shown that in natural water bodies, the concentrations of heavy metals in sediments are three to five orders of magnitude higher than in the overlaying water [2]. In soils, heavy metals may sorb on phosphate minerals, hydrous oxides of aluminum, iron and manganese [3] as well as to natural organic matter [4]. In order to enter the living cells i.e., to be bioavailable, the heavy metals have first to be solubilized, because the biological effects may only be caused by soluble ionic form of metals [5]. The term “bioavailability” is often used to describe the fraction of a substance that is actually taken up by a certain organism and can be quantified by the effect the substance has on this organism [6]. Semple *et al.* distinguished readily bioavailable and bioaccessible fractions of chemicals [7], the latter including in addition to readily bioavailable fraction also the fraction that may become available upon desorption. In this work, we have designated these fractions as **water-extracted bioavailable** (bioavailable fraction detected with sensor bacteria in soil-water extract) and **total bioavailable** (detected with sensor bacteria in soil-water suspension).

In addition to soil properties, the soil organisms themselves may greatly affect the bioavailability of heavy metals in soils: due to their direct contact with solid particles, additional (bioaccessible) fraction of heavy metals may be released from the soil or sediment [5]. Previous studies on micro-organisms have shown that only a small fraction of the total amount of a heavy metal in soil or sediment is water extractable and thus, readily bioavailable to living organism whereas the majority of heavy metals remain sorbed to soil particles [8-10]. However, experiments with recombinant metal sensor bacteria have demonstrated that a remarkably bigger fraction (even up to several orders of magnitude) of heavy metals is available to bacterial cells in soil suspensions that contain solid particles compared to particle-free soil extracts [9, 11-13]. Bioavailability and bioaccessibility of heavy metals are strongly affected by both abiotic and biotic factors. The most important abiotic factors are linked to fundamental soil properties which determine the binding of heavy metals to soil: pH, texture, aluminium, manganese and iron oxide concentration and organic matter content [14]. On the other hand, it is known that the metabolic activity of microbes influences particular soil parameters, for example pH, redox potential, ionic strength. In addition, microbes may affect metal bioavailability by biosorption, bioprecipitation, extracellular sequestration, reduction by extracellular polymers, chelators etc. [15], whereas different organisms show different effects Thus, soil microorganisms are intimately involved in metal biogeochemistry through a variety of processes determining mobility, and therefore, bioavailability. The balance between mobilization and immobilization varies depending on the organisms involved, their environment and physicochemical conditions [16].

There can be substantial differences in bacteria-metal interactions between Gram-positive and Gram-negative bacteria due to basic differences in their cell surface structures. Although it has been shown that the general cell wall construction of Gram-negative and -positive bacteria has minor influence on interactions between metals and bacterial cells [17], particular single constituents of the cell wall envelopes of these bacteria – carboxyl, amine, thiol and phosporyl groups – can greatly affect the sorption of metals onto bacterial surface [18, 19]. Additionally, some heavy metal transport proteins potentially influencing also metal bioavailability *via* their effect on heavy metal influx-efflux, occur exclusively in Gram-negative bacteria. For example, CBA transporters, three-component trans-envelope pumps, which are one of the main export mechanisms for heavy metals for Gram-negative bacteria, are poorly represented in Gram-positive bacteria due their lack of outer cellular membrane [20].

Due to a number of factors influencing the bioavailability of contaminants in soils, the determination of bioavailability is complicated and no universal chemical or biological test has been developed yet. However, there is a great need for the assessment of bioavailable fraction of metals in soils, especially for risk assessment purposes, as in most cases the actual hazard is not correlated with the chemically determined total heavy metal content – the criterion currently used for regulatory purposes. In general there are two approaches, which have been used to obtain information on bioavailability: extraction with solvents of different extraction power and subsequent chemical analysis of the obtained extracts (sequential extraction procedures; [21]) and various biological assays. Both approaches have their advantages and disadvantages. In the case of sequential extraction, correlation of chemically extracted fraction and various biological effects has still to be proven [22]. On the other hand, biological tests with certain species may not adequately predict the effects for other species of interest as it is well known that the bioavailable fraction of a chemical in a given soil or sediment can substantially differ between organisms [7].

Recombinant bacterial cells that are specially modified to respond to intracellular subtoxic concentrations of heavy metals by increasing an easily detectable signal (e.g., luminescence or fluorescence) are promising tools to detect bioavailable heavy metals [23]. A number of such recombinant bacterial sensors for the detection of Cd, As, Sb, Cr, Cu, Hg, Zn, Pb, Co and Ni [reviewed 24-26] have been developed and some of them have been used to detect bioavailable Cu [27-29], Hg [11, 12, 30] Pb and Cd [9, 11, 13, 26, 31, 32], Co and Ni [33] and As [12, 26, 34] in soil and/or sediment samples. Moreover, recently these sensors have been used to measure bioavailable metals from metal-oxide nanoparticles [35, 36]. Previous studies describing the use of recombinant metal sensor bacteria for the analysis of bioavailable metals in environmental samples have often been limited by either the number of samples (usually, the data for relatively low number of samples have been presented) or bacteria (usually one or two bacterial strains have been used). In this study we applied nine different luminescent bacterial heavy metal sensors to study bioavailability of Hg, Zn and Cd in soil.

The aims of the current study were:

- To investigate the effect of host bacterium (Gram-positive or -negative), genetic metal-response element and location of the metal-response element (plasmid or chromosome) on sensitivity (limit of determination and toxicity) of sensor bacteria towards target heavy metals
- To evaluate total bioavailable and water-extracted bioavailable fractions of Cd, Zn and Hg in spiked soils using sensor bacteria belonging to both Gram-negative (*Pseudomonas fluorescens* and *Escherichia coli*) as well as Gram-positive (*Bacillus subtilis* and *Staphylococcus aureus*) bacterial families thus representing organisms of different natural habitats, physiology and cell wall structure.
- To compare the total and water-extracted bioavailable fractions measured by different recombinant bacterial sensors in order to investigate whether the bioavailability of metals depends on the type of bacterial cell or nature of the metal-response element used for the construction of the sensors.
- Using Cd as a model, to monitor the changes in bioavailability of Cd as a result of bacterial metabolic activity during 2-hour incubation of sensor bacteria with soil-water extracts and suspensions.

## 2. Results and Discussion

### 2.1. Characterization of bacterial sensors

Different bacteria belonging to both Gram-negative (*Pseudomonas fluorescens* and *Escherichia coli*) and Gram-positive (*Bacillus subtilis* and *Staphylococcus aureu*s) groups and having different natural habitats - from soil to human gut microflora, were used as hosts for the sensors applied in this study (Table 1). However, the genetic metal-response elements (regulatory protein binding heavy metal ion(s) and its regulated promoter) used for their construction, are similar for some sensor strains thus allowing to evaluate their performance in different types of bacteria as well as interplay between the metal-response elements-mediated and bacterial physiological mechanisms-mediated mechanisms for bioavailability results. For example, **ZntR** and **P*zntA*** from Zn resistance system of the *E coli* chromosome were used in *E coli* MC1061 (pSL**zntR**/pDN**PzntA**lux) and *P. fluorescens* OS8::Kn**zntRPzntA**lux, **CadC** and ***PcadA*** from Cd resistance system in pI258 of the *Staphylococcus aureus* were used in *B. subtilis* BR151(p**cadCPcadA**lux) and *S aureus* RN4220(p**cadCPcadA**lux) and **MerR** and **P*mer*** from broad spectrum Hg resistance system of the *Serratia marcescens* plasmid pDU1358 were used in *E. coli* MC1061(p**merR_BS_BPmer**lux) and *P. fluorescens* OS8::Kn**merR_BS_BPmer**lux and OS8(pDN**merR_BS_BPmer**lux) (Table 1). In the latter-mentioned strains, in addition to regulatory protein and its regulated promoter, a gene encoding for organomercurial lyase, **MerB** is expressed. Organomercurial lyase enables the sensor to detect organomercurials in addition to inorganic mercury [37]. The elements (**MerR** and **P*mer*)** from another Hg-resistance system from Tn21 were used in fluorescent strain *E coli* MC1061(pmerGFP) [39], which was used for fluorescence microscopy.

In the case of *P. fluorescens*, metal-response elements fused with the bacterial luminescence system were expressed either in chromosomal DNA or in a plasmid (Table 1). The constructs with chromosomal insertions are genetically more stable and do not require antibiotics in the growth and test media. In this study, the plasmid-containing strains and strains with chromosomal insertions were used in parallel for bioavailability measurements of heavy metals in soils.

### 2.2. Response of the sensor strains to Hg, Cd and Zn

**Figure 1.**
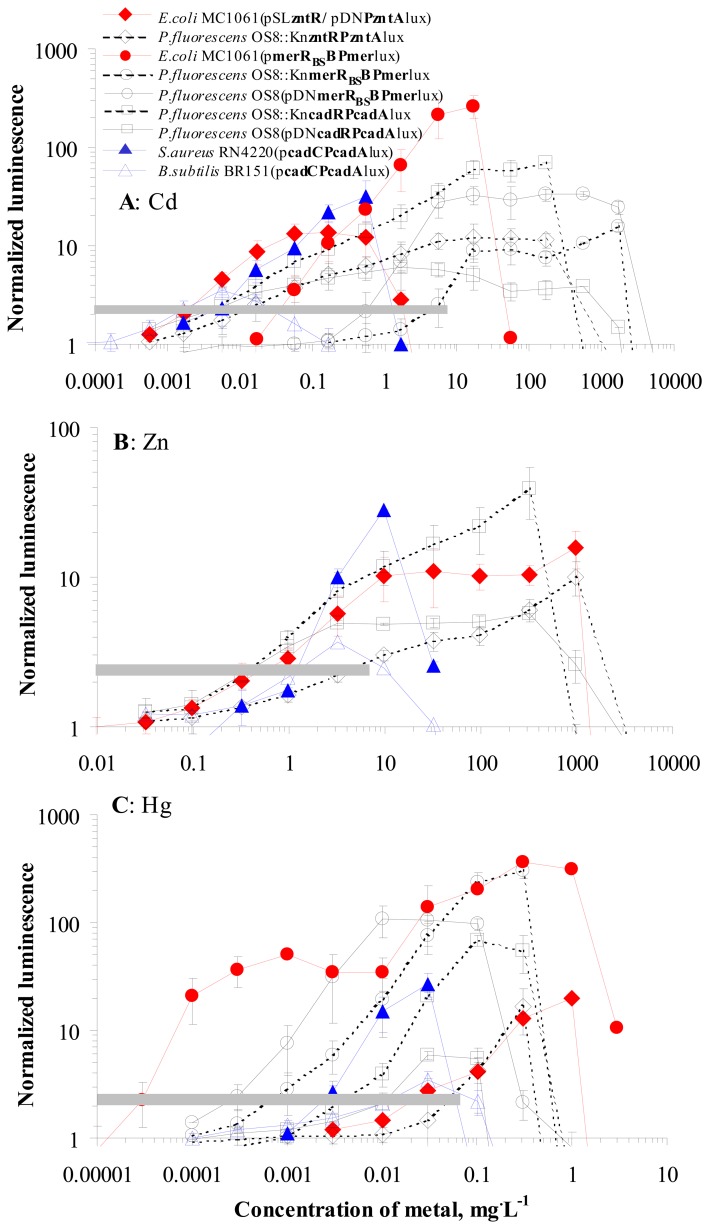
Concentration-effect curves of 9 sensor strains used in this study. Induction of luminescence (expressed as normalized luminescence) in different sensor strains by Cd (A), Zn (B) and Hg (C). Data represent mean ± standard deviation of at least three independent experiments. Dashed horizontal grey areas indicate the range of NL_LOD_ (for different sensors 2 - 4500 μg·L^-1^ of Cd , 400 - 5000 μg·L^-1^ of Zn and 0.03 - 60 μg·L^-1^ of Hg)

Induction of luminescence as well as toxic effects caused by Cd, Zn and Hg in the nine sensor strains used in this study are presented in Figure 1. All nine strains were induced by Cd and Hg and six strains were also induced by Zn. The limits of determination (LOD) of nine different sensor strains for Cd, Zn and Hg are presented in Figure 1 (range of NL_LOD_ is presented with grey horizontal lines) and as seen (the values can be read from Table 1), varied greatly for different sensor bacteria.

Analysis of the data showed that LOD values of different strains for Cd differed for three orders of magnitude: from 0.002 to 6 mg·L^-1^ (Figure 1, Table 1) and were mostly dependent on the metal-response element, expressed in the different sensor bacteria. The lowest LOD values for Cd were calculated for the sensors expressing either **CadC**/**P*cadA*** from *Staphylococcus aureus* Cd resistance system (*S. aureus* RN4220(p**cadCPcadA**lux) and *Bacillus subtilis* BR151(p**cadCPcadA**lux) ) or **CadR**/**P*cadA*** from Cd resistance system of *Pseudomonas putida* (*P. fluorescens* OS8::Kn**cadRPcadA**lux and OS8(pDNP**cadRPcadA**lux) ). The LOD values for those sensors ranged from 0.002 to 0.008 mg of Cd·L^-1^. Very low LOD (0.002 mg of Cd·L^-1^) was also obtained with the sensor *E. coli* MC1061(pSL**zntR**/pDN**PzntA**lux). In contrast, remarkably higher LOD values: 0.04 to 6 mg of Cd·L^-1^ were calculated for the strains with **MerR** and **P*mer*** (originating from relatively specific Hg resistance system) the least sensitive being *P. fluorescens* OS8::Kn**merR_BS_BPmer**lux (Figure 1A, Table 1).

Similarly to Cd, the **LOD values for Hg** differed also by about three orders of magnitude between different sensor strains (Figure 1C, Table 1) again being dependent on the metal-response element. However, for this metal the lowest LOD values were obtained for the strains expressing **MerR** and **P*mer*** (0.00003 to 0.0008 mg·L^-1^ of Hg) and the highest values (0.025 and 0.05 mg·L^-1^ of Hg) for the strains with **ZntR** and **P*zntA*** as metal-response elements.

Expectedly, comparison of the plasmid-containing and the chromosomal sensor strains of *P. fluorescens* showed that neither in the case of Cd nor Hg were there no remarkable differences in LOD values of **CadC**/**P*cadA-***expressing strains. At the same time, the plasmid-containing strain OS8(pDN**merR_BS_BPmer**lux) was 7 and 4-fold more sensitive towards Cd and Hg, respectively, than the chromosomal strain OS8::Kn**merR_BS_BPmer**lux. An explanation for this difference between the strains based on the same bacterial host and expressing similar metal-response elements, however, could not be offered on the basis of this study.

In contrast to Cd and Hg, the inter-strain variation in LOD values for Zn was only 10-fold (Figure 1B, Table 1). The lowest LOD values were obtained for sensor strains with **CadR**/**P*cadA*** from *P.putida* (OS8::Kn**cadRPcadA**lux and OS8(pDNP**cadRPcadA**lux) ) whereas the strains expressing **MerR** and **P*mer*** were not induced by Zn (data not shown).

Analogously to LOD values, the toxicity of Cd, Hg and Zn to the nine sensor strains was different. These differences were the most remarkable for Cd (about four orders of magnitude) and less for Hg and Zn (about 100-fold, Figure 1). In general, all the tested heavy metals were the most toxic to Gram-positive species, *Bacillus subtilis* and *Staphylococcus aureus. E. coli* tolerated about 30 and 20-fold and the least sensitive *P. fluorescens* tolerated about 2,500 and 40-fold higher concentrations of Cd and Zn than the Gram-positive strains, respectively (Figure 1). Thus, differently from LOD values, which were mostly dependent on the genetic metal-response element used in the sensor bacteria, the toxicity of Cd, Zn and Hg was dependent on the (bacterial) host.

It should be mentioned that the obvious correlation between the LOD values and the metal-response element used in the sensors was somewhat surprising as the bacterial species used in this study have different natural habitats (principally, with different metal concentrations): *P. fluorescens* and *B. subtilis* are common soil bacteria, *E. coli* is an enteric bacterium and *S. aureus* is an opportunistic human pathogen [40]. Therefore, these bacteria could be expected to express different heavy metal transport systems. However, our study showed that in the inducing, i.e. subtoxic concentration range, there were apparently only small differences in intracellular concentrations (resulting from the equilibrium between import and export) of Cd, Zn and Hg between the bacterial host strains used. However, some differences in LOD values were observed between the *E. coli* and *P. fluorescens* strains expressing similar metal-response elements: there was about 10-fold difference in Cd and Hg LOD values between the *E. coli* and *P. fluorescens* strains expressing **MerR** and **P*mer*** and similar difference could be seen in Cd and Zn LOD values between the *E. coli* and *P. fluorescens* strains expressing **ZntR** and **P*zntA*** (Table 1). Nevertheless, the differences in metal homeostasis in the used bacterial species become more apparent in the toxic concentrations of heavy metals: as shown above, the toxicity of Cd, Zn and Hg was evidently dependent on the host bacterium used. Toxicity of Cd, Hg and Zn for the used bacterial hosts decreased in the order *B. subtilis* = *S. aureus* < *E. coli* < *P. fluorescens* and markedly, **Gram-positive bacteria were more sensitive to those heavy metals than Gram-negative**. Higher observed toxicity of the tested divalent cations to Gram-positive bacteria could be explained by the ability of Gram-negative bacteria to produce glutathion, which mediates tolerance to several transition metals [41].

### 2.3. Bioavailability of Cd, Zn and Hg in soil

**Scheme 1.**
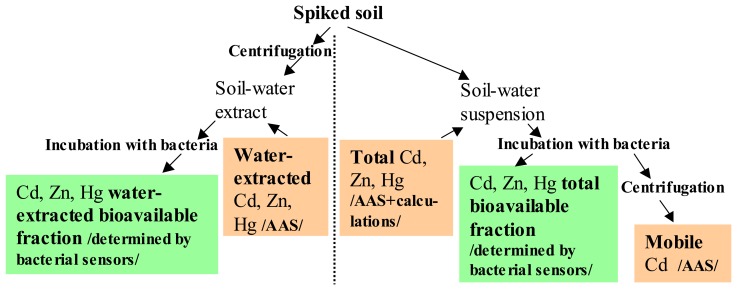
Schematic depiction of the current study.

Nine heavy metal sensor strains: recombinant *Pseudomonas fluorescens*, *Escherichia coli*, *Bacillus subtilis* and *Staphylococcus aureus* were used to study the bioavailable fractions of Cd, Zn and Hg, frequent pollutants from various industrial activities, in soil. Both, soil-water extracts and the respective soil-water suspensions were analyzed with sensor bacteria to determine the **water-extracted bioavailable** (determined in soil-water extracts) and **total bioavailable** (determined in soil-water suspensions) fractions of these metals (see Scheme 1 in Materials and Methods). As discussed above, the bacterial species used in this study, have different natural habitats, physiology and presumably also different metal homeostasis. Microbial physiology and heavy metal homeostasis are the most important factors influencing the toxicity of heavy metals to microbial cells. Indeed, microbial cells may influence their surrounding environment and thus, also affect bioavailability and speciation of heavy metals [15].

As a study matrix, a presumably clean agricultural soil was selected and spiked with different concentrations of Cd, Zn and Hg. Before spiking, the concentrations of Cd and Hg in the soil were very low: 0.45 and 0.14 mg·kg^-1^, respectively. Also, the concentration of Zn (219 mg·kg^-1^) did not exceed the limit values for soils according to Council Directive 86/278/EEC. Other characteristics of the soil used for spiking are described in Materials and Methods. Spiking was chosen as a method as this allows to control the sample properties while changing only the nature and concentration of the studied metal. Indeed, in our previous paper we demonstrated that among 60 soils with different structure and composition, Cd bioavailability varied up to two orders of magnitude depending mainly on the soil type [9]. Moreover, the use of controlled physico-chemical conditions in the soil enables to investigate the differences in bioavailability of heavy metals to different bacterial species carrying different metal-response elements. The non-spiked soil did not induce bioluminescence in any sensor strain used for testing and thus, was used as a control soil to take into account the non-specific effects of the soil on bacterial bioluminescence. Indeed, the soil-water suspension was causing a remarkable decrease in the bacterial bioluminescence (from 74 to 92%; data not shown). As the decrease of luminescence by soil suspension was relatively similar for all the bacterial strains, it could be assumed that quenching of bioluminescence was an optical shading effect due to soil particles. The soil-water extract however did not have almost any effect on bacterial bioluminescence. As an average, only 4% decrease (i.e., average CF = 1.04) in bacterial background bioluminescence was observed when analyzing soil-water extracts.

Bioavailability of Cd, Zn and Hg in the studied soil to 9 different bacterial strains is presented in Table 2. In this soil, Zn and Cd were the least bioavailable metals. Only 0.24 – 0.37 % of the total Zn and 0.19 – 0.46 % of the total Cd was detected in soil-water extract by recombinant sensors (i.e. was water-extracted bioavailable) (Table 2). Despite of similar low water-extractability of Cd and Zn, their bioavailability in soil-water suspension was different. When sensor bacteria were incubated in contact with soil solid particles (i.e., total bioavailability was analyzed) 7-21 fold (14-fold as average for all sensor strains) more **Cd** was bioavailable than in soil-water extract indicating that the direct contact between bacterial cell and soil matrix had a remarkable effect on Cd bioavailability. In general, the difference in total and water-extracted bioavailability of Cd was similar for all the bacteria proposing that the mechanisms, playing a role in the elevated bioavailability, most probably due to the release of additional Cd from soil solid particles, were similar. The reasons for elevated bioavailability of Cd in the case of direct contact between soil and bacterial cells require further studies. However, it could be assumed that one of the reasons could be the production of certain exudates like weak acids [42], extracellular polysaccharides or chelators (reviewed in [15]) by bacteria and thus, the change in the equilibrium between metal complexation and dissociation in soil matrix. Also bacterial biosurfactants have been assumed to have an effect on heavy metal bioavailability [43,44].

Differently from Cd, bioavailability of Zn in soil-water suspension remained in the same range as in soil-water extracts (0.32 – 0.61 % of total) suggesting that all the bioavailable Zn, although a relatively small fraction, was in soluble form and readily bioavailable to bacteria in soil-water extracts.

**Figure 2.**
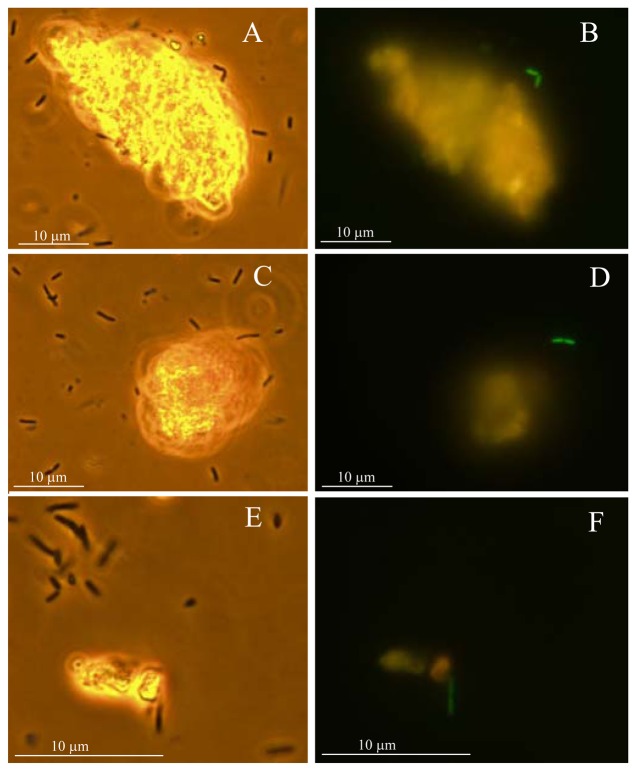
Induction of GFP in Hg sensor *Escherichia coli* MC1061(pmerRGFP) in soil-water suspensions of Hg-spiked sample 11 (characteristics of the sample are given in Table 4 in Materials and Methods). Photos were taken by using phase-contrast light microscopy (A, C, E) or by fluorescence microscopy (475/515 nm) (B, D, F) after 8-hour induction of *E. coli* MC1061(pmerRGFP). A and B, C and D, E and F show similar views by light or fluorescence microscopy. Green cells (B, D, F) indicate the presence of bioavailable Hg.

In accordance with [11], Hg was the most extractable and bioavailable metal in the studied soil. As average for all the used sensor strains, 3.3 % of the total Hg was bioavailable in soil-water extract and 3.2-30.5% of Hg was bioavailable in soil-water suspension, depending on type of the sensor bacteria: the bioavailability of Hg in soil suspension assays was about 10-fold higher to Gram-negative sensor strains than to Gram-positive ones whereas the latter were only accessing the water-extracted fraction of Hg (Table 2). This result indicates that bioavailability of heavy metals in soil depends both on heavy metal as well as on the type of bacterial cell. For Gram-negative sensor strains however a fraction of particle-bound Hg become apparently bioavailable upon direct bacteria-soil contact most probably due to the altered complexation-dissociation of this metal in these conditions. To visualize the effect of solid particles on Hg bioavailability, we used a fluorescent sensor *E. coli* MC1061(pmerGFP) [39], in which the GPF is induced by Hg-response elements (**MerR** and **P*mer*)** from Tn21 (Table 1). After incubation of these sensor bacteria with the water-suspension of Hg-spiked sample no 11 (Table 4 in Materials and Methods) and observing individual bacteria in fluorescence microscope, we showed that the green fluorescent signal was remarkably more induced in the sensor cells attached to or located in the vicinity of soil particles than in the sensor cells moving freely between soil solid particles (see Figure 2A and B, C and D, E and F).

**Figure 3.**
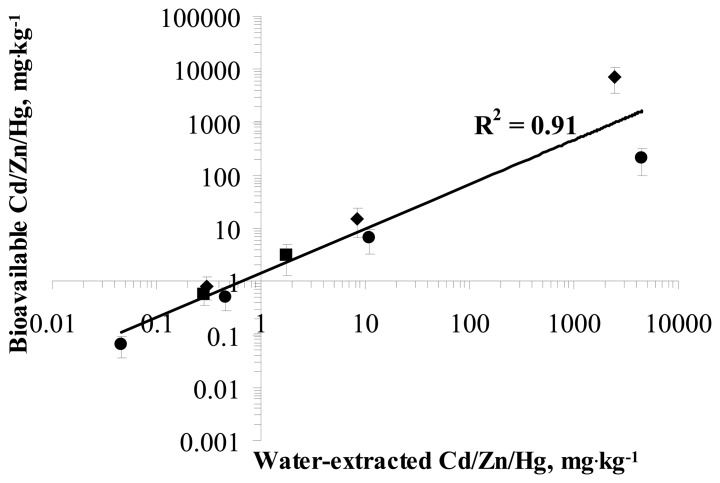
Correlation between water-extracted and bioavailable Cd, Zn and Hg in the soil samples 1-13. Water-extracted (determined from water extracts of soil samples 1-13 by AAS) fractions of Cd (●), Hg (■) and Zn (♦) plotted against respective bioavailable (measured by heavy metal sensor bacteria; fractions. R^2^ of correlation between water-extracted and bioavailable fractions of Cd, Zn and Hg is presented.

It is interesting to note that the water-extracted bioavailable concentrations of Zn, Cd and Hg measured by the sensor bacteria were almost equal to and showed very good correlation (R^2^= 0.91) with the total concentrations of those metals measured in soil-water extracts by AAS (Figure 3). These results showing 100% bioavailability of water-extracted metals are in agreement with previous studies showing that water-extracted Cd from soil was fully bioavailable to bacteria [9-10, 13]. Also, 100% bioavailability of water-soluble As and Pb to bacteria has been reported earlier [9, 13, 34, 44].

### 2.4. Differences in bioavailability of Cd, Zn and Hg in soil to different sensor bacteria

Comparison of the results of water-extracted bioavailability of Cd (0.14 – 0.46% of total), Zn (0.24 – 0.37% of total) and Hg (1.9 – 2.6% of total) measured by 9 different sensor bacteria showed that there were no statistically significant differences between different sensor strains. This was not surprising as in the soil-water extract most of the metals could be expected to be in the form of free solubilized ions and hence, bioavailable to enter the bacterial cells. Indeed, the bioavailable and total concentrations of Zn, Cd and Hg in soil-water extracts were practically identical (Figure 3).

Comparison of the results on total bioavailability (measured in soil-water suspensions) of Cd and Zn (Table 2) showed that similarly to soil-water extracts there were no statistically significant differences between different sensor strains. This result is somewhat surprising as in the case of direct contact between the soil and test bacterium, the bacterial cells could be expected to influence certain soil parameters and thus, also metal bioavailability. Indeed, our results showed that the direct contact between the bacterial cells and the soil matrix caused partial release of particle-bound fraction of Cd and allowed its entrance into bacterial cells. However, this additionally released fraction of Cd was similar for all the tested bacterial strains and not dependent on the specific properties of bacterial cell (Table 2).

There was a significant difference (*p* < *0.01* according to single-factor ANOVA) between the total bioavailability of Hg measured by the Gram-negative and the Gram-positive sensor strains in soil-water suspension (Table 2). Differential bioavailability of Hg depending on the Gram-staining of the host bacterium is interesting and might thus be due to differences in cell wall composition. Higher uptake of mercury by Gram-negative bacteria could lead to higher toxicity of this metal in natural ecosystems as it has been shown that Gram-negative bacteria are more frequently present e.g., in sediments [45]. Moreover, in anaerobic sediments, the relatively high bioavailability of Hg to Gram-negative bacteria could lead to bacteria-mediated mercury methylation [46] and increased probability to enter the food chain.

Taking together, apart from Hg in soil-water suspensions, bioavailability of the tested metals was similar for all nine bacterial strains differing from each other either by host bacterium or the genetic metal-response element. This result is especially interesting as the concentrations of metals inducing different sensors were remarkably different, particularly in the case of Cd and Hg (Figure 1) and shows that sensor bacteria with various metal-response elements could be used interchangeably for measuring bioavailable heavy metals. However, attention should be drawn to the type of bacterial host used: according to the results of this work, the representatives of both Gram-negative as well as of Gram-positive group should be included in the analysis of bioavailability of heavy metals to soil microflora.

### 2.5. Time-dependent changes in bioavailability of Cd in soil

**Figure 4.**
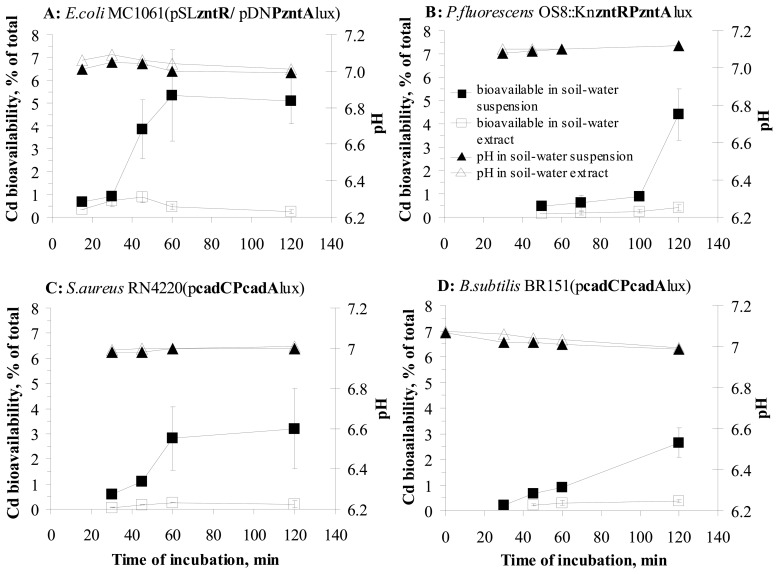
Bioavailability of Cd in water suspensions and the respective extracts of Cd-spiked soils to different recombinant sensor bacteria during 2-hour incubation. Bioavailability of Cd in soil-water suspension (■) or soil-water extract (□) determined by different sensor bacterial strains (A-D). pH in soil-water suspension (▲) or extract (Δ) during 2-hour incubation is presented.

To study the effect of incubation time on the release of particle-bound metals from soil solid matrix, we studied the time-dependent changes in water-extracted and total bioavailable fractions of Cd – a metal with similar water-extracted and total bioavailability for all the nine sensor strains (Table 2) in samples 1-5 (see Table 4 in Materials and Methods). Bioavailability of Cd in those soils was measured by two Gram-negative bacterial strains: *E. coli* MC1061(pSL**zntR**/pDN**PzntA**lux) and *P. fluorescens* OS8::Kn**cadRPcadA**lux and two Gram-positive strains: *B. subtilis* BR151(p**cadCPcadA**lux) and *S. aureus* RN4220(p**cadCPcadA**lux). As the standard incubation time for all the previous measurements was two hours, we were interested on changes in bioavailability before the 2-hour measurement point to map the early changes and kinetics of Cd desorption. However, it should be mentioned that the induction of different strains by Cd started at different time-scale and thus, bioavailable amount of Cd could not be calculated for all the strains in very early measurement points. For *B. subtilis* BR151(p**cadCPcadA**lux) bioavailable Cd could be calculated starting from 30-minute incubation and for *S. aureus* RN4220(p**cadCPcadA**lux) and *P. fluorescens* OS8::Kn**cadRPcadA**lux only after 45-and 50-minute incubation (Figure 4B, C and D).

Results on changes in water-extracted and total bioavailability during 2 -hour incubation as well as pH during that period are presented in Figure 4. The pH of the test environment did not practically change during the incubation period and only a 0.2 unit decrease was observed in soil-water suspensions and extracts when incubated with *E. coli* cells. No change or very small changes in pH could be explained by the high buffering capacity of M9 medium (phosphate buffered medium at pH 7.1). However, despite the very small change in pH, significant changes in bioavailability of Cd in soil-water suspension to all 4 sensor bacteria during 2-hour incubation were observed.

The water-extracted bioavailable Cd fraction did not change practically in soil-water extracts during 2-hour incubation for any of the bacterial strains used (Figure 4); only a slight increase in the water-extracted bioavailable Cd concentration (Figure 4B, C) was observed for *P. fluorescens* and *B. subtilis* (2.3 and 2.8-fold compared to the results obtained after 30, 45 and 50-minute incubation). This result was somewhat expected as our previous studies showed nearly 100% bioavailability of water-extracted Cd (Figure 3).

However, in soil-water suspensions bioavailability of Cd increased considerably during 2-hour incubation for all the bacterial strains used. Compared to the earliest measurement points where the bioavailability could be calculated, Cd bioavailability in soil-water suspension increased 13-fold for *S. aureus* RN4220(p**cadCPcadA**lux), 7.6-fold for *E. coli* MC1061(pSL**zntR**/pDN**PzntA**lux), 5.5-fold for *P. fluorescens* OS8:: Kn**cadRPcadA**lux and 4.6-fold for *B. subtilis* BR151(p**cadCPcadA**lux) (Figure 4). For *B. subtilis* BR151(p**cadCPcadA**lux) and *P. fluorescens* OS8:: Kn**cadRPcadA**lux bioavailability of Cd was also determined after 3-hour incubation with soil-water suspensions, however no additional increase of Cd bioavailability was observed (data not shown). Moreover, the amount of bioavailable Cd for *B. subtilis* and *P. fluorescens* even decreased between 2 and 3-hour incubation: 1.2 fold and 1.9-fold, respectively.

The data showing only slight increase in Cd bioavailability in (particle-free) soil-water extracts but considerable increase in bioavailable Cd in soil-water suspensions (contact assay) suggest that during 2-hour incubation with soil-water suspension, the bacterial cells cause desorption of certain fraction of particle-attached metal in soil, which is not extracted with water. Similar effect has also been demonstrated in other papers showing that organisms can influence the biologically accessible fraction of organic compounds in soil by changing the compound's mass transfer rate [47]. However, the mechanisms facilitating the increased uptake of metals to bacteria in the case of direct soil-bacteria contact, need to be elucidated.

In parallel to bioavailability assays, we measured the amount of Cd mobilized by *E. coli* MC1061(pSL**zntR**/pDN**PzntA**lux), *P. fluorescens* OS8::KncadRPcadAlux, *B. subtilis* BR151(p**cadCPcadA**lux) and *S. aureus* RN4220(p**cadCPcadA**lux) from soil-water suspensions after different exposure times (see Scheme 1 in Materials and Methods). Interestingly, although the bioavailability of Cd in soil-water suspension increased over time (Figure 4), no remarkable increase in mobile Cd (measured by AAS from soil-water extracts after centrifugation of bacteria-exposed soil-water suspensions) was observed (Table 3). Thus most probably the bacterial activity had an effect on heavy metal bioavailability only in microenvironments and did not affect the mobilization of Cd in large scale.

## 3. Materials and Methods

### 3.1 Bacterial strains

The recombinant sensor strains used in this study have been constructed earlier and are based on *Echerichia coli* MC1061 (*araD*139 Δ(*ara*, *leu*)7697 Δ*la*cX74 *galU galK hsdR*2 *strA mcrA mcrB*1), *Pseudomonas fluorescens* OS8 (Rif^r^, isolated from soils polluted with toluates) [47], *Staphylococcus aureus* RN4220 (*r*sbU-,agr-) and *Bacillus subtilis* BR151 (*trpC*2 *lys*-3 *metB*10). The strains contain different genetic heavy metal response elements (Table 1) controlling the expression of either *luxCDABE* genes of *Photorhabdus luminescens*[48] or GFP. The luminescent bacterial strains used in this study were constructed by Ivask and Rõlova [49] and were used in this study to quantitatively detect bioavailable heavy metals in water-suspensions and extracts of soils. The fluorescent Hg sensor *E. coli* MC1061(pmerRGFP) was from [38] and used for fluorescence microscopy. In the case of *P. fluorescens*, the metal-response elements are either in a plasmid or incorporated into the bacterial chromosome (Table 1).

### 3.2. Cultivation of bacteria

Sensor bacteria were grown overnight in LB medium (3 mL; per litre: 10 g of tryptone, 5 g of yeast extract, 5 of g NaCl) [50] supplemented with 20 μg·L^-1^ of tetracycline [OS8(pDN**cadRPcadA**lux) and OS8(pDN**merR_BS_BPmer**lux)], 100 μg·L^-1^ of kanamycin [OS8::Kn**cadRPcadA**lux, OS8::Kn**zntRPzntA**lux, OS8::Kn**merR_BS_BPmer**lux)], 30 μg·L^-1^ of kanamycin [BR151(p**cadCPcadA**lux), RN4220(p**cadCPcadA**lux)], 100 μg·L^-1^ of ampicillin [MC1061(p**merR_BS_BPmer**lux) and MC1061(pmerGFP)] or 100 μg·L^-1^ of ampicillin and 10 μg·L^-1^ of tetracycline [MC1061(pSL**zntR**/ pDN**PzntA**lux)]. M9 medium (10-50 mL; per liter: 6 g Na_2_HPO_4_, 3 g KH_2_PO_4_, 0.5 g NaCl, 1 g NH_4_Cl, 0.25 g MgSO_4_×7H_2_O, 0.01 g CaCl_2_; pH 7.0) [50] supplemented with glucose (final concentration 2 g·L^-1^) and acid hydrolysed casein (final concentration 5 g·L^-1^) was inoculated with 1/50 diluted overnight culture and bacteria were grown until mid-exponential phase, OD_600_ of 0.6. Optical density was measured with spectrophotometer Jenway 6300 (Spectronic Analytical Instruments, Garforth, UK). Amount of viable bacterial cells in the test was determined by spreading the sensor bacterial culture prior the test on LB agar plates supplemented with appropriate antibiotics and growing them at 30°C for 24 hours.

### 3.3. Soil samples and their preparation

Uncontaminated sandy soil (containing 10.6% of clay, 10.6% of silt, 72.8% of sand, 5.7% of organic matter; 39 g·kg^-1^ of CaCO_3_, 3.59 g·kg^-1^ of N, 0.62 g·kg^-1^ of P; with 2.3 cmol^+^ kg^-1^ of CEC and pH 7.3) from northwestern Estonia whose Cd, Zn and Hg content is shown in Table 4 was used. The soil was spiked with different concentrations of HgCl_2_ CdCl_2_×2H_2_O and ZnCl_2_ as described earlier [11] to obtain 13 spiked soil samples with different heavy metal concentrations. Initial (natural background) Cd, Zn and Hg concentrations of this soil (determined in a certified laboratory) as well as concentrations of Cd, Zn and Hg added to the soil to obtain 13 spiked soil samples are presented in Table 4. The sums of the initial amounts of metals in soil and the added amounts were considered as the total amounts of Zn, Cd and Hg in spiked soils. Non-spiked soil was included in each assay as a control to determine the general effect of soil (e.g., quenching of light) on bacterial bioluminescence in the test.

For bioavailability analysis, soil-water suspensions and extracts were prepared by mixing of 1 g of dry soil with 12.5 mL of MilliQ water and rotating at room temperature for 24 h (see Scheme 1).

After this equilibration, soil-water suspensions were obtained and soil-water extracts were further prepared by centrifugation of the soil-water suspensions at 13,000 g for 5 minutes and separation of the supernatant. Water-extracted heavy metals were measured from soil-water extracts by AAS in a certified laboratory (Scheme 1). Each soil sample was prepared and analyzed at least in three replicates.

### 3.4. Bioavailability tests and calculations

Bioavailability of heavy metals in the soils was calculated either for soil-water suspensions (**total bioavailability** to sensor bacteria) or the respective soil-water extracts (**water-extracted bioavailability** to sensor bacteria) (see Scheme 1). Water (100 μL), heavy metal solution with suitable concentration, soil suspension or soil extract was pipetted in two parallels onto white 96-well Cliniplates (Thermo Labsystems, Helsinki, Finland). Into each well, an equal volume (100 μL) of the sensor bacteria (as suspension in the growth medium) was added, and the plates were incubated for 2 h without shaking at 30^o^C (optimal time for the induction and synthesis of luciferase – a reporter protein). Luminescence was measured with microplate luminometer Labsystems Fluoroskan (Thermo Labsystems, Helsinki, Finland). If instead of the microplate cuvettes were used, the luminescence was measured with tube luminometer 1253 (Thermo Labsystems, Helsinki, Finland). In order to determine the changes in Cd bioavailability in time, incubation times different than 120 minutes (2 h): 15, 30, 45, 50, 60, 70, 100, 120, 180 and 195 minutes, were used for selected sensor strains. All the induction measurements were performed in duplicate. At least two (in most cases three) independent experiments were performed in order to calculate the standard deviations. pH values in the test was measured with an Orion ROSS Combination microelectrode (ThermoScientific, USA).

Calculations were done according to [51]. Response of sensor bacteria to heavy metal standards was calculated by following formula:

(1) $$NL=\frac{L_{S}}{L_{B}}$$

where NL is normalized luminescence (showing fold-induction of luminescence in sensor by bioavailable metals), L_S_ is the bioluminescence (in RLU – relative light units) measured in a metal-containing sample, and L_B_ a bioluminescence (in RLU) measured in water (background luminescence). In the case of soil-water suspensions and extracts the effect of soil matrix on bacterial bioluminescence was taken into account. For that, correction factor CF was calculated as follows:

(2) $$CF=\frac{L_{B}}{L_{CS}}$$

where L_CS_ is bioluminescence (in RLU) of sensor bacteria in suspension or extract of non-spiked (control) soil. In water suspensions and extracts of spiked soils, the induction of sensor bacteria was calculated by taking into account the CF as follows:

(3) $$NL=\frac{L_{s}}{L_{B}}\times CF$$

In parallel to each assay with soil-water suspensions or the respective extracts, heavy metal standard solutions were analysed with the sensor bacteria in order to build standard calibration curve (built for every incubation time where bioavailability was measured) later used to calculate bioavailability. Water solutions of HgCl_2_ CdCl_2_×2H_2_O and ZnSO_4_×7H_2_O [analytical grade (98%), Riedel-de-Häen (Seelze, Germany)] were used as standards. The limit of determination (LOD) was determined in each measurement and was defined as the minimal concentration of certain heavy metal (in water solution) that caused the lowest detectable induction (NL_LOD_) of the corresponding sensor calculated as follows:

(4) $$NL_{\text{LOD}}=2\frac{{\bar{L}}_{B}+3S_{D}}{{\bar{L}}_{B}}$$

where ${\bar{\text{L}}}_{\text{B}}$ is the mean background luminescence of at least four parallels and S_D_ is their standard deviation. LOD (as mg·L^-1^ of metal) was calculated from the linear regression of standard curve constructed by plotting of log_10_ values of NL and metal concentration.

Bioavailable Cd, Zn and Hg was calculated on the basis of the constructed standard curve and the induction (>NL_LOD_) of bioluminescence by the sample.

Bioavailability data for different bacterial strains were compared by using single factor analysis of variance (ANOVA). A statistical difference was considered significant when *p* <0.01.

### 3.5. Fluorescence microscopy

The strain *Escherichia coli* MC1061(pmerGFP) [39] was used to study the induction of fluorescence by bioavailable Hg under fluorescence microscope. Bacteria were grown as described above and incubated with suspension of soil sample 11 (Table 4) for 8 hours at 37 **°**C in an Eppendorf tube. Bacteria-soil mixture (5 μL) was transferred from the bottom of the tube to the microscope slide, covered, incubated for 20 minutes and visualized by fluorescence microscope Olympus CX41, filters 475/515 nm and 1,000-fold magnification. In parallel, photos were taken at similar magnification by phase contrast light microscopy (Olympus CX41).

### 3.6. Measurement of mobile Cd from soil

To determine the concentration of mobile Cd, 1:10 soil-water suspensions incubated for 0.5, 1 or 2 h with equal amount of bacterial suspension (prepared analogously to the bioavailability test, see above), were centrifuged at 13 000 g for 5 minutes. The concentration of Cd in the resulting soil-water extracts was measured by AAS (Shimadzu, Kyoto, Japan).

## Conclusions

In this study, we calibrated and used nine different recombinant luminescent metal sensor bacteria belonging to both Gram-negative (*Pseudomonas fluorescens* and *Escherichia coli*) as well as Gram-positive (*Bacillus subtilis* and *Staphylococcus aureus*) genera to analyze the water-extracted bioavailable (measured in soil-water extracts) and the total bioavailable (measured in soil-water suspensions) fractions of Cd, Zn and Hg in soil. The following conclusions could be drawn on the basis of this study:

- The limit of determination of the sensors was determined mainly by the type of the genetic metal-response element used for the construction of the sensor bacteria. At the same time, toxicity of the Cd, Zn and Hg standard solutions was mostly dependent on the host bacterium, Gram-positive bacteria being in general more sensitive to all the metals than Gram-negative.
- Bioavailability of Cd, Zn and Hg in soil did not depend neither on the limit of determination (determined according to standard calibration curve) of the used sensor nor on the metal-response elements expressed in these sensor cells.
- The water-extracted bioavailable fractions of Zn, Cd and Hg were low (making 0.24 – 0.37%, 0.19 – 0.46 % and 1.7 – 4.9 % of the total Zn Cd and Hg, respectively) and similar to all the used sensor strains.
- The total bioavailable fraction of Cd and Zn (2.6 – 5.1% and 0.32 – 0.61%, of the total Cd and Zn, respectively) was almost comparable for all the sensors whereas the bioavailability of Hg in soil-water suspensions was about 10 fold higher for Gram-negative sensor cells (30.5% of total Hg) compared to Gram-positive ones (3.2% of the total Hg).
- In the case of Zn, the water-extracted and total bioavailable fractions were equal indicating that no additional Zn could be mobilized by the sensor bacteria upon direct contact with soil matrix in suspension assay.
- The bioavailable fraction of Cd and Hg (only in the case of Gram-negative sensor strains) in soil-water suspensions exceeded the water-extracted bioavailable fraction about 14-fold indicating that upon direct contact, additional fraction of Cd and Hg was mobilized by those sensor bacteria
- Using Cd as a model we showed that in the used test conditions, 2-hour incubation (standard incubation time for the test with sensor bacteria) was enough for all the used bacterial strains to access all potentially available Cd in the soil-water suspensions.

Thus, according to the results of this study, for robust bioavailability studies of heavy metals in soils any type of genetic metal response elements could be used for the construction of the sensor strains. However, there might be differences between Gram-negative and Gram-positive bacteria groups and thus, respective strains should be used in parallel if testing bioavailability of metals in the environment.

## Figures and Tables

**Table 1.** Recombinant bacterial strains used in this study and their inducibility with heavy metals.

**Host bacterium and sensor strain**	**Genetic metal-response element a**	**Location of metal-response elements**	**Inducing metals b**	**Limit of determination (LOD), μg·L^-1^±SD**		

				**Cd^2+^**	**Zn^2+^**	**Hg^2+^**
**Gram-negative**						
***Pseudomonas fluorescens*OS8**						

OS8::Kn**cadRPcadA**lux c	CadR/P*cadA*	chromosome	Cd, Zn, Hg,Pb	7 ± 2.5	400 ± 110	4 ± 1.3
OS8(pDN**cadRPcadA**lux)^d^	CadR/P*cadA*	plasmid	Cd, Zn, Hg,Pb	8 ± 1.4	500 ± 40	15 ± 1.7
OS8::Kn**zntRPzntA**lux^c^	ZntR/P*zntA*	chromosome	Cd, Zn, Hg,Pb	20 ± 4.7	5000 ± 580	60 ± 19
OS8::Kn**merR_BS_BPmer**lux^c^	MerB/MerR/P*mer*	chromosome	Hg, MeHg, Cd	4500 ± 1230	not induced	0.8 ± 0.2
OS8(pDN**merR_BS_BPmer**lux)^d^	MerB/MerR/P*mer*	plasmid	Hg, MeHg, Cd	650 ± 220	not induced	0.2 ± 0.05
***Escherichia coli* MC1061**						

MC1061(pSL**zntR**/pDN**PzntA**lux)^d^	*ZntR*/*PzntA*	plasmid	Cd, Zn, Pb	2 ± 0.5	700 ± 170	20 ± 5
MC1061 (p**merR_BS_BPmer**lux)^d^	MerB/MerR/P*mer*	plasmid	Hg, MeHg, Cd	40 ± 13	not induced	0.03 ± 0.009
MC1061(pmerGFP)^f^	MerR/P*mer*	plasmid	Hg^e^			0.6^e^

**Gram-positive**						
***Bacillus subtilis*BR151**						

BR151(p**cadCPcadA**lux)^d^	CadC/P*cadA*	plasmid	Cd, Zn, Hg,Pb	2 ±0.3	1000 ± 150	10 ± 1.5
*Staphylococcus aureus***RN4220**						

RN4220(p**cadCPcadA**lux)^d^	CadC/P*cadA*	plasmid	Cd, Zn, Hg,Pb	7 ± 2	1500 ± 210	2 ± 0.7

a (complementary gene)/regulatory protein/regulated promoter

b from a tested set of metals comprising of Cd, Zn, Hg, methylmercury, Pb, Cu, Ag. Tests with Pb were carried out in HMM medium lacking inorganic phosphates, tests with other metals were done in M9 (see Materials and Methods)

c constructed by T. Rõlova [38]

d constructed by A. Ivask [38]

e strain constructed and data from [39]; not tested with other metals than Hg MeHg –methylmercury

**Table 2.** Bioavailability of Cd, Hg and Zn in soil to different sensor strains. Data represent mean ± standard deviation of three independent experiments.

**Metal**	**Host bacterium (species)**	**Strain**	**Metal-response elements (location)^a^**	**Viable cells in test**	**Total bioavailable b,%of total**	**Water-extracted bioavailable^c^,%of total**
**Cd^d^**	***Gram-negative***					
	*Pseudomonas fluorescens*	OS8::Kn**cadR PcadA**lux	CadR/P*_cadA_* (C)	3×10^7^	3.5 ± 1.8	0.46 ± 0.19
		OS8(pDN**cadR PcadA**lux)	CadR/P*_cadA_* (P)	4×10^6^	4.4 ± 2.5	ND
		OS8::Kn**zntR PzntA**lux	ZntR/P*_zntA_* (C)	6×10^6^	4.8 ± 0.7	0.23 ± 0.0011
		OS8::Kn**merR_BS_B Pmer**lux	MerR/P*_mer_* (C)	2×10^7^	2.6 ± 0.4	0.41 ± 0.039
		OS8(pDN**merR_BS_B Pmer**lux)	MerR/P*_mer_* (P)	5×10^6^	3.7 ± 1.5	ND
	*Escherichia coli*	MC1061(pSL**zntR**/ pDN**PzntA**lux)	ZntR/P*_zntA_* (P)	1×10^7^	5.1 **+** 0.51	0.24 ± 0.18
		MC1061(p**merR_BS_B Pmerl**ux)	MerR/P*_mer_* (P)	4×10^7^	3.7 ± 1.5	0.44 ± 0.18

		**AVERAGE for Gram-negative bacteria**			**4.2±0.71**	**0.36±0.11**

	*Gram-positive*					
	*Bacillus subtilis*	BR151(p**cadC PcadA**lux)	CadC/P*_cadA_* (P)	3×10^6^	3.2 ± 1.1	0.19 ± 0.08
	*Staphylococcus aureus*	RN4220(p**cadC PcadA**lux)	CadC/P*_cadA_* (P)	8×10^6^	2.6 ± 0.3	0.38 ± 0.054

		**AVERAGE for Gram-positive bacteria**			**2.9±0.36**	**0.28±0.13**

**Hg e**	*Gram-negative*					
	*Pseudomonas fluorescens*	OS8::Kn**cadR PcadA**lux	CadR/P*_cadA_* (C)	3×10^7^	27.0 ± 7.7	2.4 ± 0.73
		OS8(pDN**cadR PcadA**lux)	CadR/P*_cadA_* (P)	4×10^6^	28.1 ± 14.0	ND
		OS8::Kn**zntR PzntA**lux	ZntR/P*_zntA_* (C)	6×10^6^	26.7 ± 1.1	2.6 ± 0.16
		OS8::Kn**merR_BS_B Pmer**lux	MerR/P*_mer_* (C)	2×10^7^	31.9 ± 12.2	2.6 ± 0.58
		OS8(pDN**merR_BS_B Pmer**lux)	MerR/P*_mer_* (P)	5×10^6^	18.7 ± 7.3	ND
	*Escherichia coli*	MC1061(p**merR_BS_B Pmerl**ux)	MerR/P*_mer_* (P)	1×10^7^	27.9 ± 5.3	1.9
		MC1061(pSL**zntR**/ pDN**PzntA**lux)	ZntR/P*_zntA_* (P)	4×10^7^	38.9 ± 5.6	1.67 ± 0.40

		**AVERAGE for Gram-negative bacteria**			**30.5±5.17**	**2.2±0.43**

**Hg e**	*Gram-positive*					
	*Bacillus subtilis*	BR151(p**cadC PcadA**lux)	CadC/P*_cadA_* (P)	3×10^6^	3.8 ± 2.8	4.9 ± 1.3
	*Staphylococcus aureus*	RN4220(p**cadCPcadA**lux)	CadC/P*_cadA_* (P)	8×10^6^	2.6 ± 1.2	2.8 ± 1.3

		**AVERAGE for Gram-positive bacteria**			**3.22±0.81**	**3.92±1.51**

**Zn^f^**	*Gram-negative*					
	*Pseudomonas fluorescens*	OS8::Kn**cadR PcadA**lux	CadR/P_cadA_ (C)	3×10^7^	0.35 ± 0.17	0.34 ± 0.18
		OS8(pDN**cadR PcadA**lux)	CadR/P_cadA_ (P)	4×10^6^	0.32 ± 0.045	ND
		OS8::Kn**zntR PzntA**lux	ZntR/P_zntA_ (C)	6×10^6^	0.42 ± 0.19	0.37 + 0.11
	*Escherichia coli*	MC1061(pSL**zntR**/ pDN**PzntA**lux)	ZntR/P_zntA_ (P)	4×10^7^	0.61 ± 0.35	0.27 ± 0.037

		**AVERAGE for Gram-negative bacteria**			**0.36±0.05**	**0.37±0.04**

	*Gram-positive*					
	*Bacillus subtilis*	BR151(p**cadC PcadA**lux)	CadC/P_cadA_ (P)	3×10^6^	0.44 ± 0.20	0.24 ± 0.11
	*Staphylococcus aureus*	RN4220(p**cadCPcadA**lux)	CadC/P_cadA_ (P)	8×10^6^	0.33 ± 0.18	0.25 ± 0.0018

		**AVERAGE for Gram-positive bacteria**			**0.39±0.07**	**0.25±0.004**

a regulatory protein binding heavy metal/promoter regulated by that protein

b in soil-water suspension (see Scheme 1 in Materials and Methods)

c in soil-water extract (see Scheme 1 in Materials and Methods)

d Average bioavailability for soils 1-5 (Table 4 in Materials and Methods)

e Average bioavailability for soils 9-13 (Table 4 in Materials and Methods)

f Average bioavailability for soils 6-8 (Table 4 in Materials and Methods)

ND – not determined

P - plasmid

C - chromosome

**Table 3.** Mobilization of Cd from soil-water suspensions by test bacteria after different exposure times. The values show mobilized^a^ Cd in μg·L^-1^.

**Bacterium**	**Time of incubation, min**			

	**0**	**30**	**60**	**120**
*Pseudomonas fluorescens* 0S8::Kn**cadRPcadA**lux	1.7	2.21	2.19	2.21
*Escherichia coli* MCI061 (pSL**zntR**/pDN**PzntA**lux)	1.7	3.18	3.20	3.19
*Staphylococcus aureus* RN4220(p**cadCPcadA**lux)	1.7	2.60	2.50	2.56
*Bacillus subtilis* BR151 (p**cadCPcadA**lux)	1.7	2.70	2.85	3.41

a mobilized Cd was measured from extracts (see Scheme 1 in Materials and Methods) of soil-water suspensions after different exposure times with bacteria.

**Table 4.** Heavy metal concentrations in the soil and spiked samples.

**Soil**	**Cd**	**Zn**	**Hg**

		**Total, mg·kg^1^dwt of soil**		

Non-spiked soil^a^	0.45	219	0.14

Spiked soil samples		**Added metal^b^, mg·_kg**^-^**^1^dwt of soil**	
1	1.5	0	0
2	15	0	0
3	150	0	0
4	1500	0	0
5	15000	0	0
6	0	900	0
7	0	9000	0
8	0	90000	0
9	0	0	0.28
10	0	0	2.8
11	0	0	17
12	0	0	28
13	0	0	280
	**Permitted limit values for soil^c^**		

	1-3	150-300	1-1.5

a used for spiking of soils 1-13 (containing 10.6 % of clay, 10.6 % of silt, 72.8 % of sand, 5.7 % of organic matter; 39 g·kg^-1^ of CaC0_3_, 3.59 g·kg^-1^ of N, 0.62 g·kg^-1^ of P; with 2.3 cmol^+^ kg^-1^ of CEC and pH of 7.3

b Amount added to the soil (in addition to its natural background heavy metal content); HgCl_2_, CdCl_2_×2H_2_0 and ZnCl_2_ were used for spiking

c According to Council Directive 86/278/EEC on the protection of the environment, and in particular of the soil, when sewage sludge is used in agriculture
